# Variation in RAD51 details a hub of functions: opportunities to advance cancer diagnosis and therapy

**DOI:** 10.12688/f1000research.15650.2

**Published:** 2018-12-03

**Authors:** Nick LL van der Zon, Roland Kanaar, Claire Wyman

**Affiliations:** 1 Department of Molecular Genetics, Erasmus MC, Rotterdam, PO Box 2040, 3000 CA, The Netherlands; 2 Oncode Institute, Erasmus MC, Rotterdam, PO Box 2040, 3000 CA, The Netherlands; 3 Department of Radiation Oncology, Erasmus MC, Rotterdam, PO Box 2040, 3000 CA, The Netherlands

**Keywords:** Homologous recombination, BRCA2, variants of unknown significance, nucleoprotein complexes, structural biology, cancer

## Abstract

Loss of genome stability is one of the hallmarks of the enabling characteristics of cancer development. Homologous recombination is a DNA repair process that often breaks down as a prelude to developing cancer. Conversely, homologous recombination can be the Achilles’ heel in common anti-cancer therapies, which are effective by inducing irreparable DNA damage. Here, we review recent structural and functional studies of RAD51, the protein that catalyzes the defining step of homologous recombination: homology recognition and DNA strand exchange. Specific mutations can be linked to structural changes and known essential functions. Additional RAD51 interactions and functions may be revealed. The identification of viable mutations in this essential protein may help define the range of activity and interactions needed. All of this information provides opportunities to fine-tune existing therapies based on homologous recombination status, guide diagnosis, and hopefully develop new clinical tools.

## Introduction

Homologous recombination (HR) is essential for the maintenance of genome stability. By taking advantage of DNA sequence complementarity, HR is uniquely suited to repair a variety of DNA lesions—such as DNA double-strand breaks (DSBs) and stalled or collapsed DNA replication forks—that affect both strands of the double helix ^1^. A key intermediate in the complex choreography of the DNA strands underlying HR is single-stranded DNA (ssDNA), generated near the lesion, bound by RAD51 protein. Initial nucleation of RAD51 on the ssDNA is followed by binding of additional protomers, resulting in a dynamic nucleoprotein filament with homology search and DNA strand exchange capabilities ^2^. The product of the breast cancer–associated gene 2 ( *BRCA2*) acts as a molecular scaffold (mediator) in this process by chaperoning RAD51 onto replication protein A (RPA)-coated ssDNA ^3^. Although HR is required for normal cell function, genome sequencing approaches ^4^ and functional HR assays performed on viable tumor material ^5^ reveal that HR deficiency is more widespread in tumors than originally anticipated. This insight is important because patients with HR-deficient tumors can benefit greatly from treatment with poly ADP-ribose polymerase (PARP) inhibitors, which selectively kill HR-deficient cells ^6^. Because worldwide sequencing efforts of germline and tumor DNA identify more and more variants of RAD51, assessing the functional consequences of these amino acid variations is of utmost importance for prognosis, diagnosis, and precision therapies for patients with cancer. This review will provide the latest advances and insights gained on the functional implications of RAD51 mutations.

The biochemical and biological functions of RAD51 can be related to the structure of individual protomers, the filaments they form, and their dynamic filament properties. It has long been appreciated that the particular structural features of filaments formed by recombinase proteins like RAD51 reflect its function. Recombinase nucleoprotein filaments are arranged with proteins in a right-handed helix around DNA. Functionally important parameters, such as the number of monomers per turn of the helix, the helical pitch, and rise per monomer, define these nucleoprotein filaments ^7^. More detailed structural information is available on the interface between monomers, which comprises the ATPase active site of DNA-bound recombinases ^8^. Additional dynamic properties such as kinetic parameters for filament assembly and disassembly as well as continuity and flexibility have more recently been defined on the basis of single-molecule methods ^9^. These all proved to be rich quantitative characteristics of wild-type RAD51 for comparison with any new variants and predicting the effect of amino acid changes.

## RAD51 nucleoprotein filaments: variety in form and function

Bacterial RecA is the paradigm recombinase first described in detail to define the filament structure. Nucleoprotein filament structures of RecA and RAD51 have been reconstructed from negative stain electron microscope images. This revealed, among other features, that both proteins form filaments with a variety of conformations ^7^. The first atomic resolution crystal structure of a eukaryotic nucleoprotein filament, yeast Rad51, revealed that the protomer–protomer interface created an ATPase active site ^8^. This nucleotide-binding protomer interface was in two different conformations. Specifically, the movement of two amino acid side chains, Phe187 and His352, resulted in a rigid body movement of one protomer relative to the other, creating the two conformations ^8^. That structure was solved for a version of Rad51 missing 79 N-terminal amino acids. Subsequent crystal structures of full-length Rad51, including these N-terminal amino acids, showed only one conformation for the ATPase interface ^10^. Although there were other differences in the proteins from these two studies, it is possible that the N-terminus constrains the filaments to a single conformation at the ATPase interface ^8, 10^. More recently, high-resolution structural models of human RAD51 filaments have been obtained from cryo–electron microscopy reconstructions ^11, 12^. Here, filaments formed with human RAD51 had only one conformation at the nucleotide-binding site. However, there is evidence that this region influences conformation at the dimer interface and filament dynamics. It has been shown that interaction of the N-terminal domain of RAD51 with the BRC4 peptide of BRCA2 at the protomer–protomer interface restricts RAD51 dynamics, locking it into a single conformation in the filament ^13^. Together, the structural studies of yeast Rad51 with and without the N-terminal 79 amino acids and the effect of BRC4 interaction with the N-terminus of human RAD51 show a role for these regions in filament arrangement and dynamics. Control and tuning of dynamic variation at the protomer contacts in the filament are certainly important aspects of RAD51 catalyzed homology recognition and DNA strand exchange functions.

More globally, RAD51 nucleoprotein filaments can be described by helical parameters and dynamic rearrangements. Specifically, active filaments formed with ATP or an analog bound at the interface have a longer helical pitch and an “open” conformation ^12, 14^. Recently, RAD51 nucleoprotein filaments with distinct mechanical properties have been defined for the wild-type protein (for example, persistence length, helical pitch, and rise per monomer) by a combination of single-molecule force spectroscopy and crystallography ^15^. One form is described as the “open” or “extended” conformation, which displays a helical pitch of 134 Å and a rise per monomer of 18.4 Å. This conformation is presumed to be the active state required for strand exchange. The second—considered to be the “closed”, “condensed”, or “collapsed” conformation—displays a helical pitch and rise per monomer of 112 Å and 16.9 Å, respectively. This difference results in a change of the monomers per helical turn of 7.3 versus 6.6 for the “open” versus “closed” conformation, respectively ^15^. These two forms differ in position of a hinge at the protomer interface, resulting in tilting of one protomer relative to the other in the “closed” conformation with respect to the “open” conformation. Because the measured energy difference between these two forms was small, they could readily switch between the two conformations by thermal excitation, independent of other energy input or ATP hydrolysis ^15^. These data lead to the conclusion that RAD51 nucleoprotein filaments can interconvert between two different conformational states. The functional role of this intriguing dynamic interchange has yet to be defined but is consistent with larger filament dynamics associated with ATP hydrolysis, which has already been linked to function ^16, 17^. Flexible and dynamic arrangements of filament structure are likely to be important, even essential, to the RAD51 catalyzed function: homology recognition and DNA strand exchange. Recent work has modified the ATPase active site to see what effect this has on filament form and function. Specifically, filaments formed with RAD51 mutants, K113A and K113R, which are located in the ATPase domain, have been characterized. RAD51 K131A does not bind ATP, and RAD51 K113R can bind but not hydrolyze ATP. Both mutants form filaments with a “closed” conformation, as does the wild-type protein with the non-hydrolyzable ATP analog AMP-PNP ^12^. It is suggested that these forms, with different helical pitch and rise, could represent intermediate steps in dynamic filament assembly and rearrangement important for function.

Like many other proteins, RAD51 is modified by phosphorylation in response to genotoxic stress. The importance and biochemical effect of the several phosphorylation sites have been difficult to sort out (as discussed in Subramanyam *et al*. ^18^). Clear effects of one RAD51 phosphorylation site, Y54, at the protomer interface were obtained by mimicking phosphorylation with a non-natural amino acid (p-carboxymethyl-L-phenylalanine, or pCMF). RAD51 with Y54 replaced by pCMF has enhanced DNA strand exchange activity. The enhanced activity is coupled to weaker DNA binding and formation of less stable/more dynamic filaments, emphasizing the essential role of dynamic interactions among RAD51 itself and DNA to perform its core biochemical function ^18^. Another example illustrating the importance of the RAD51–RAD51 interface is demonstrated by using small-molecule chemical inhibitor RI-1 ^19^. This inhibitor covalently targets C319. C319 is located at the protomer–protomer interface, where it serves as an ATP cap, overlaying the nucleotide-binding site. The inhibitor thereby disrupts the protomer–protomer interface ^19^. RAD51 filaments apparently have a “plastic” structure, and interconvertible conformations are influenced by the state of nucleotide bound at their interface, specific amino acid interactions, post-translational modifications, and chemical compounds. Mutations that potentially affect RAD51 filament dynamics will be discussed in the next section.

## RAD51 mutations associated with human disease

**Figure 1.**
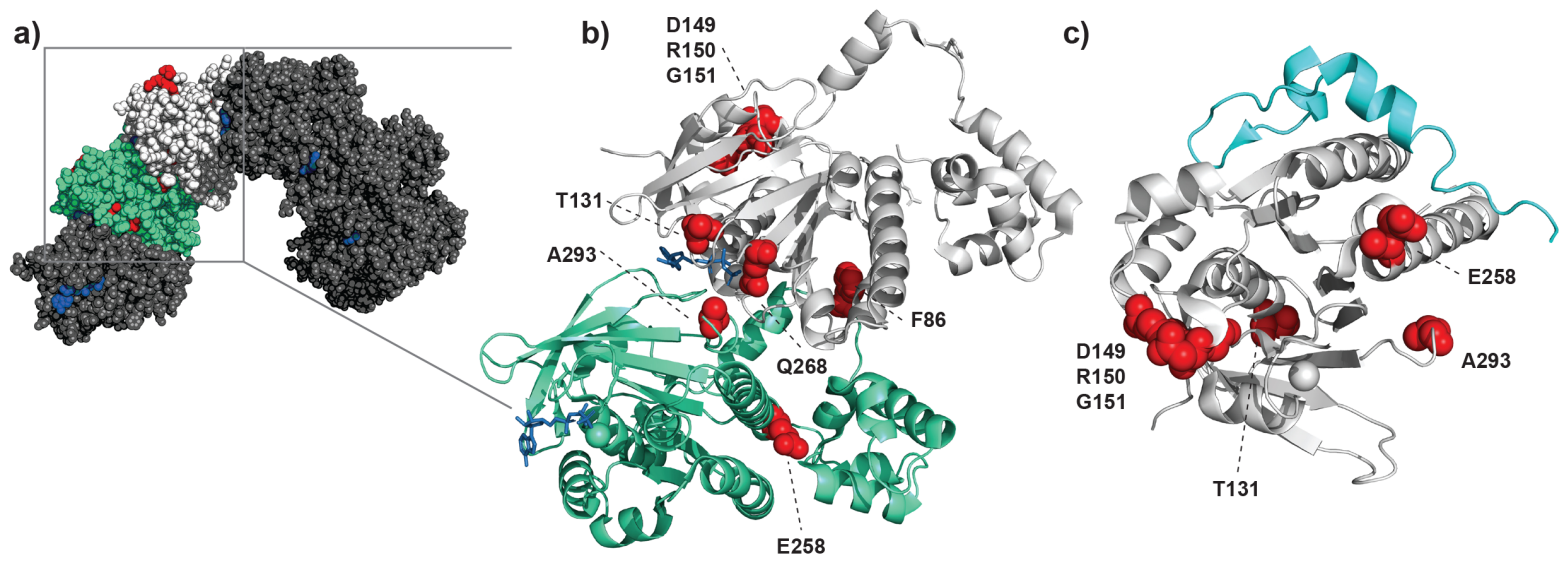
Overview of RAD51 structures and interactions. Overview of RAD51 mutations mapped onto structures based on PDB ID 5NWL ^15^ and 1N0W ^26^. ( **a**) Model based on the crystal structure of a heptameric right-handed RAD51 nucleoprotein filament. Two adjacent protomers are colored green and light gray; the other five protomers are shown in darker gray. ATP is highlighted by the blue areas at the interfaces of distinct protomers, and sites of mutation are highlighted in red. The red patch on the top of the white-colored protomer is the patch of amino acids 149–151 that is assumed to affect interaction of RAD51 with other binding partners. Note that Q272 is not highlighted in the figure, as that residue was not resolved in the crystal structure. ( **b**) Focus on the interface between two protomers, where ATP is shown in blue and the discussed sites of mutation are highlighted with red spheres. Most of these mutations can be found at the direct interface between the two protomers and thus close to the nucleotide-binding pocket. ( **c**) Interaction of RAD51 (light gray) with a BRCA2 BRC repeat (cyan). The locations of mutation discussed are highlighted in red. Here, one mutation is in close proximity of the binding region with the BRC repeat (E258) and therefore might impair RAD51 binding by BRCA2.

Because RAD51 nucleoprotein filament formation and dynamics are essential elements of its DNA strand exchange function, small changes to the protomers could cause dramatic effects. Mutations affecting DNA binding, filament formation or structure, ATP hydrolysis, or strand exchange would all influence function in HR. Here, we will specifically discuss recently identified mutations and what is known about their effects on RAD51 function ( Table 1 and Figure 1). New RAD51 mutants have been discovered from functional HR assays on tumor material, genome sequencing of patients with phenotypes like those of Fanconi anemia (FA) (a genetic disease including HR defects), and large-scale cancer genome sequencing. The mutations causing FA-like phenotypes are perhaps the easiest to understand functionally. They are T131P and A293T, located at the ATPase active site interface, close to the amino acids F129 and H294, equivalent to F187 and H352 in yeast Rad51. These amino acids are expected to influence the conformation at this interface and its alternating conformations, which they directly contributed to in yeast Rad51 filaments ^8, 12^. Mutations creating different chemical environments in close proximity of this interaction might affect conformational dynamics of human RAD51 nucleoprotein filaments. For example, A293T at the protomer–protomer interface is directly adjacent to a conserved loop involved in DNA binding and might explain the impaired filament assembly and stability ^25^. These mutations have a dominant negative effect in some biochemical assays ^21, 25^.

**Table 1.** Recently identified RAD51 mutations.

Mutation	Sort of mutation	DNA-binding activity	ATPase activity	Strand exchange activity	Reference(s)
*F86L*	Somatic	Impaired	Normal	Impaired	20
*T131P*	Germline	Normal	DNA-independent	Impaired	21
*D149N*	Somatic	Impaired	Normal	Normal	22
*R150Q*	Germline	Impaired	Impaired	Normal	22
*G151D*	Somatic	Impaired	Impaired	Enhanced	22, 23
*E258A*	Germline	Impaired	DNA-independent	Impaired	20
*Q268P*	Somatic	Impaired	Normal	Impaired	24
*Q272L*	Somatic	Impaired	Normal	Impaired	24
*A293T*	Germline	Impaired	Impaired	Impaired	25

Genome analysis of patients with cancer has revealed RAD51 somatic mutations with interesting functional implications. Changes in three adjacent residues (D149, R150, and G151) located in the so-called Schellman loop were identified in breast cancer ^22^. This region is reported to include a p53-binding site ^27– 29^. These three adjacent amino acids map onto RAD51 at a position distinct from the DNA-binding or ATPase interface sites ^22^. The last of these three mutations, G151D, does have altered DNA-binding and ATPase activity, indicating that filament architecture or dynamics or both are affected ^23^. Mutations found in kidney and lung tumors, Q268P and Q272L, are located in a DNA-binding loop important for allosteric activation of ATP hydrolysis and DNA strand exchange ^24^. Additional breast cancer–associated mutations F86L and E258A are located in the multimerization interface of RAD51 nucleoprotein filament. The DNA-binding loop and interface mutations, like the mutants described above, have a dominant negative effect in biochemical assays for RAD51 function ^20, 24^. Because RAD51 is an essential protein, these viable mutants may identify a functional range of activity. This aspect will be important to better define if such changes are to be targeted for eventual therapeutic intervention. Interestingly, F86L and E258A change the RAD51 protomer interface as well as the interaction interface with BRCA2 ^20^. This example points out that RAD51 mutations not only impair nucleoprotein filament structure or stability but also can affect interactions with other partner proteins such as BRCA2 or possibly p53. BRCA2 is also an essential protein and its presumed essential role is as a RAD51 chaperone ^3, 4^. Our own work indicates that all diffusing nuclear RAD51 is bound to BRCA2, indicating that even minor changes to structure at the interface may have large consequences ^30^.

## The possible effects of RAD51 mutations on the interaction with BRCA2

The recent analysis of RAD51 we have reviewed focused mainly on the effects on filament formation and function. Nucleoprotein filament dynamics and activities, essentially RAD51–RAD51 and RAD51–DNA interactions, are not the only interactions needed in normal HR function. Interaction partners, such as BRCA2, are obviously also needed for proper HR. BRCA2 is a large protein (3,418 amino acids) that includes a central region with eight BRC repeats that bind RAD51 and at least one additional RAD51-binding motif at the C-terminus. BRCA2–RAD51 interaction is needed to transfer RAD51 to replace RPA on ssDNA originating from the DSBs ^31, 32^. Partial structures of a complex including one BRC repeat and RAD51 are available ^26, 33^. The interface between RAD51 and BRCA2–BRC repeats overlaps with the RAD51–RAD51 interaction. Thus, changes in RAD51–RAD51 interface may also affect its interaction with BRCA2 ( Figure 1C). Additional binding sites of RAD51 are present in BRCA2, such as the C-terminal domain of BRCA2, which is suggested to bind across a RAD51–RAD51 interface in the nucleoprotein filament ^34^. The importance of interaction between RAD51 and BRCA2 is demonstrated by observations that mutated BRC repeats conferred DNA-damage sensitivity *in vivo* ^35^ and altered RAD51 nucleoprotein filament formation *in vitro* ^36– 38^. The importance of RAD51 mutations on its interaction with additional partners and mediators, such as the RAD51 paralogs PALB2, MMS22L-TONSL, RADX, RAD54, RAD51AP1, and others ^39, 40^, awaits structural information on their relevant interfaces. The RAD51 mutations we have reviewed may indicate important additional interactions or identify the dynamic range of interaction strengths tolerated for RAD51 function in cell viability. Altered RAD51 is linked to cancer and other diseases affected by impaired genome stability. A detailed definition of RAD51 mutations linked to functions, emerging from the work we review here, is needed to guide personalized anti-cancer therapies and guide diagnosis based on variants of currently unknown significance.

## Abbreviations

BRCA2, breast cancer–associated gene 2; DSB, double-strand break; FA, Fanconi anemia; HR, homologous recombination; pCMF, p-carboxymethyl-L-phenylalanine; RPA, replication protein A; ssDNA, single-stranded DNA.
